# How sulfadoxine-pyrimethamine (SP) was perceived in some rural communities after phasing out chloroquine (CQ) as a first-line drug for uncomplicated malaria in Tanzania: lessons to learn towards moving from monotherapy to fixed combination therapy

**DOI:** 10.1186/1746-4269-2-5

**Published:** 2006-01-10

**Authors:** Stephen ED Nsimba

**Affiliations:** 1 Department of Clinical Pharmacology, Muhimbili University College of Health Sciences (MUCHS), Dar-es-Salaam, Tanzania

## Abstract

Malaria is a leading cause of death in Sub-Saharan Africa. Tanzania changed its malaria treatment policy from chloroquine (CQ) to Sulphadoxine-Pyrimethamine (SP) as first line drug in August 2001. We wanted to assess the perception and behaviour about SP after phasing out chloroquine which was very popular, cheap, available, and was preferred by many people for self-medication in homes as it was considered to have minimal side effects.

Focus Group Discussions (FGDs) were carried out after one year of the anti-malarial drug treatment policy change in the country. The FGD themes were on malaria for under-five children and other age groups, anti-malarial drug use through self-medications, specific experiences people had about SP drug for both mothers/guardians, men in the communities and health workers. A total of twelve FGDs were performed with mothers/guardians, men and health workers in the selected public health care facilities in the district.

In the FGDs people feared adverse reactions from SP; its slow ability of reducing fever and self-treatment in this case was less reported from the mothers/guardians groups. However, SP was reported by health workers to be administered using the direct observation approach under supervision in their health care facilities. This was done in order to increase compliance as there were worries that some mothers were throwing away the drug if they were instructed by health workers to go and administer SP to their sick children at home.

Based on this information, it is obvious that fear and negative perceptions about SP drug was common in the study setting. As evidence of this, there was less reported home-stocking and self-medication in the discussions for this particular recommended new first-line anti-malarial. The public has demonstrated a lack of confidence in SP. Furthermore, some health workers expressed obvious fear and negative perceptions towards the drug despite the fact that some FGDs with health workers considered the drug to be good and effective against malaria. Such negative perception towards SP highlights the need to start earlier sensitization and educational campaigns to the rural communities for a new drug program to ensure its success. Messages should clearly state what should be expected from the new drug (Coartem), before its introduction. This is important especially as Tanzania is expected to move towards the expensive but efficacious and effective fixed-combination (Coartem) anti-malarial therapy early next year (2006).

## Introduction

Worldwide, malaria causes more than 1.5–3 million deaths each year of which more than 90% occur in under-five children in sub-Saharan Africa [1]. The majority of those are among the children who have not developed adequate immunity to the parasite and also in pregnant women. However, malaria is on the increase due to changes in the environment, the collapse of health systems in areas of civil strife and war, growing resistance of malaria to affordable anti-malarial drugs and limitations in national health services.

Despite many attempts to eradicate or control malaria, the disease still threatens about 40% of the world populations (300–900 million cases every year). Malaria is endemic in almost all parts of Tanzania, and it accounts for over 30–40% of the disease burden (admissions and outpatient attendances). It is a major cause of under-five mortality and morbidity. About 70,000–100,000 under-five deaths which occur annually in the country are due to malaria [2]. Malaria is a threat to every one as it affects all age groups but under-fives, pregnant women and non-immune individuals are more vulnerable than others. The cause of death in these vulnerable groups is mostly due to cerebral complications and anaemia especially in under-fives children and pregnant women. Malaria is also a major contributor to maternal deaths and low birth weights of children [3].

Chloroquine (CQ) has been used for many years as first line treatment drug for uncomplicated malaria in many sub-Saharan countries including Tanzania. Nevertheless, because of rapid development and spread of resistance to the drug in African countries, these countries have been forced to change their anti-malarial drug treatment policies to sulfadoxine-pyrimethamine (SP). However, it is very unfortunate that even to this newly recommended drug (SP) resistance is reported to be developing very fast in most places of sub-Saharan countries [4,5]. So far when Tanzania changed its anti-malarial drug treatment policy to SP in August, 2001, chloroquine resistance was more than 42% [6].

The widespread self-medication practices for most febrile illnesses is a known major problem in sub-Saharan countries [7]. Such irrational self-treatment practice is reported to be accompanied with either over-or-under-dosages [8], with an increased risk of toxicity or emergence and widespread development of drug resistance [9]. Furthermore, SP is reported to be expensive in terms of price when compared to chloroquine in Tanzania [10].

Tanzania is a country heavily affected by malaria, as it combines widespread prevalence of the malaria mosquito with low levels of government and community resources available to combat the disease. Over 75% of Tanzania's 35 million people live in areas where malaria is highly endemic (transmission period longer than 4 months per year), and the estimated number of cases is between 14–18 million annually. Next to the enormous loss of productivity due to illness and the costs of treatment, malaria results in an estimated 100,000 to 125,000 deaths per year, of which 70% are children under the age of five [2]. For Tanzania, malaria is not just a health issue, but a national development problem.

Tanzania has made malaria control a priority and is an active partner in the global Roll Back Malaria (RBM) partnership, set up by the WHO in 1998. Comprehensive malaria control policies have been formulated by the National Malaria Control Program, a large number of pilot projects have been implemented, national first-line drug treatment for malaria was changed in August, 2001 to Sulfadoxine-Pyrimethamine (SP) to overcome chloroquine resistance. Despite these efforts, much remains to be done as local governments and poor rural communities lack resources to implement successful malaria control programs.

Thus, it was necessary to study how SP was perceived by both the mothers/guardians, men in the communities and health workers who are entrusted with providing care to these communities within their catchment areas. SP was introduced after the government (Ministry of Health) decided to phase out chloroquine from being used as first-line-anti-malarial for treatment of uncomplicated malaria for all age groups in Tanzania. The aim of this study was to access people's perceptions, compliance, self-medication practices (rational use of SP), household responsibilities in case of sickness and source(s) of information about SP. This study also aimed at forming basis for instituting interventions to improve compliance and rational drug use practices which may help in prolonging the life span of the available first line anti-malarial drug in the country. Such rational practices if achieved would also increase the cure rates and reduce treatment failures.

Furthermore, Tanzania will early next year (2006) be replacing SP with an expensive but efficacious and effective fixed-anti-malarial-combination of artemether and lumefantrine therapy (Coartem). This new fixed combination drug will need its life span be preserved so that it remains in use for at least the same duration or more than what chloroquine served over 40 years in Tanzania.

## Materials and methods

### Study area

The study was conducted in Kibaha district, Coast region-Tanzania. The district is located 40 km north-west of Dar es Salaam, Tanzania. The district was chosen because malaria is endemic to holoendemic. Furthermore, Kibaha is also geographically accessible from Dar-es-Salaam the economical capital of the country. According to the National Census of 2002, Kibaha district has a total population of 132,443 people [11].

### Selection of study population

A three-stage cluster sampling procedure was used in the selection of wards, hamlets and households. Our sampling framework was based on the administrative structure of the Kibaha district. In Tanzania, all districts have divisions, and divisions are subdivided into wards. In urban areas, wards were further subdivided into hamlets (*vitongoji*), whereas, in rural settings, wards were subdivided into villages and the villages were further subdivided into hamlets.

The district had a total of 9 wards and of these 4 were urban and 5 were rural. After listing all the wards, then two rural and two urban wards were randomly selected. A list of the villages and hamlets in the chosen wards was obtained from the District Planning Officer (DPO).

A total of 12 focus group discussions consisted of 6–10 participants each were held, four with mothers, four with men and four with health workers. The participants in the mother's/men focus groups were chosen with help from the ward secretaries and the district nursing officer for the health worker focus groups. The mothers and men and health workers were aged between 18–50 years. Most of them had completed primary school education. The health workers FGD groups were divided according to their educational level and their training background. Thus, making one group of clinical officers and assistant clinical officers (COs & ACOs)), one group of trained nurses and two groups consisted of nurse assistants.

### Data collection

#### Focus group discussions (FGDs)

Guided themes for the FGDs were developed using the *Swahili *language (National language spoken by most Tanzanians) and were pre-tested before carrying out the actual study. The themes included self-medication practices with SP and other different anti-malarials, perceptions toward SP, any untoward experiences with SP or rumours they have heard about the drug and from which source. The discussions were held in undisturbed locations with one moderator who was an experienced social scientist from the Department of Sociology, University of Dar-es-Salaam. He was assisted by the PI and the main author (SN). Note taking was done by two graduate social scientists who had just completed their Masters of Arts in Sociology at the University of Dar-es-Salaam. All discussions were tape recorded and later on transcribed from the Swahili version into English. The translation into English included also the Swahili hand written notes. This translation was done by the author (SN) and one of the note taker (DS).

The FGD approach was used for data collection because it allows the researchers to get ideas about peoples experience, opinions, beliefs and participants' responses can be presented with actual quotes that helps the reader to get the main ideas or messages. Furthermore, it gives opportunity to observe the interactions within the groups which is normally lacking with interviews.

### Data analysis

The focus group discussions were analysed using a combination of qualitative approaches or methods [12] which also included coding and structuring the data into categories [13]. Furthermore, analysis also used the traditional interpretive understanding approach [14]. This analysis is a process of making sense of the collected information through eliciting meanings and responses the study participants gave during the FGDs. A code sheet was created following the focus group guide and data were coded. Then a master sheet was done giving listing of all the responses from the focus groups. These responses were interpreted by looking at their patterns. Finally sorting and sifting through information, looking for patterns, consensus, differences, variations or contradictions and weighing the relative importance of information complimented the interpretative understanding. Raking and tabulation of the data was also done.

### Ethical Approval

Ethical approval was obtained from the human ethics committees of the Muhimbili University College of Health Sciences and from the Kibaha district, Coast Region administrative authorities, ward secretaries, village chairmen and leaders of the hamlets.

## Results

### Knowledge about "SP"

Focus group discussions (FGDs), revealed some knowledge about the drug (SP) and it's use. Some participants reported that malaria parasites were resistant (in a *Swahili *word [*sugu*] (meaning resistant) to CQ. The mother's FGDs reported "*sisi tunalazimishwa tukutunia hii dawa ya SP kwa sababu klorokwini haipo kwenye maduka ya dawa*" [our translation] they are forced to use SP in public health facilities and CQ is not available in drug stores or shops. Some said "*sisi tungependa kuendelea kutumia klorokwini kama ingekuwa ni hiari ya mtu kuchagua kwani bado tunaamini inaweza kutibu malaria*" [english translation] they would have liked to continue using CQ if they were given freedom to chose as they strongly believed CQ was still effective for malaria treatment (Tables 1 &2).

**Table 1:** Shows summaries of statements made by different focus group discussions (4 FGDs) with mother's/guardian's about SP

**About SP**	**Why switch to SP?**	**How can the situation be improved?**	**Who's responsibility to care the sick child?**	**What's wrong with the media?**
- SP is mixture of all drugs like Fansidar. - SP is not good because it causes: swelling, body rashes, children dying. - We are afraid of using it because it is too strong and kills people. - My son became like a cobra-snake and got black like a charcoal after using SP. - SP has no side-effects to me and when I use it cures my malaria. -SP is a good drug, my son used it and has never fallen sick again. - The drug is too strong and one needs to eat enough food. - People are afraid of using the drug and we hate it and we don't give our children when they give us at the hospital. - Now we are being forced at the hospital to give our children the drug under supervision of nurses. -Parasite are resistant to CQ.	-SP is on research trial by the government. - They want to see how many people will be killed by the drug. - If drug was good, we would not find it plenty in our hospitals. - We have nothing to do, but agree with what the government gives us as we are forced to use the drug. - The Ministry of Health should work hard on sensitizing and educating the people. - If you tell the doctors that you don't use SP he/she may listen to you and give an alternative drug or not it depends. - We are treated with SP at the facilities for all illnesses. - Doctors don't ask whether don't use SP.	- More research has to be done how to lower the dose and what causes these side effects. - Educate people first about SP before introducing for large scale use. - Nurses should educate mother's on how to take this drug and about side effects to be expected. - Educate the news writers about the drug. - The sulfa component should be removed from SP. - We want doctors and nurses to properly inform us how to use SP whether without eating etc - Nurses and doctors should be given seminars on how to properly treat malaria. - Doctors should listen carefully to patients so that they can reduce the dose as the drug is too strong for weak people. - Put in place drugs which are less strong for mild to moderate malaria and reserve SP for severe cases.	- When the children are sick we mothers take them to hospital. - Father's directs us not to take SP. - Father work to get money and most of the time they are out but they provide money. - If the father is not at home and the child is sick I can't wait, I go myself to hospital or take any other immediate actions. - We mother's are first child's doctors in our homes. - Some men are lazy and sometimes they are drunk and do not care about the child. - Most fathers' get involved when the child is seriously ill. - At times we discuss with our husband's if they are at home, otherwise we brief them what transpired when they were not around. - In most cases we involve them if the child's condition gets worse. - The father mostly gives you money to go and buy drugs or as bus fare to send the child to hospital and very rarely they escort you.	- SP is advertised so much in newspapers, radio and television. - They show people who gets affected by the drug after using it. - They also put a name and a picture of a person who has been affected by SP. - These news scare a lot of people who see or read them. - We also here in radio that chloroquine is no longer working in treating malaria.

**Table 2:** Shows summaries of statements made by different focus group discussions (4 FGDs) with men about SP

**About SP**	**Why switch to SP?**	**How can the situation be improved?**	**Who's responsibility to care the sick child?**	**What's wrong with the media?**
- People who had experienced side effects due to SP can never use the drug again in their life time. -Most people fear using SP because they think it is not a good drug. health facilities because people don't use the drug may be unless you force them -When one takes the drug needs to eat a enough food and drink a lot of water -Few people get side effects due to SP and actually it is not easy to tell if really what people complain is due to SP. -A lot of people are affected by and many have died. -Children who use SP get very weak, tired, and some become confused. - In general SP cures better than chloroquine and it is a good drug except of its minor problems it causes -SP is a combination of Fansidar, Metakelfin and Septrin.	-Even if you tell the doctor that you don't have malaria still you will get SP prescribed. - These drugs are still on trial and this is bad to play with peoples lives. - Sometime when you ask the doctors about SP they become harsh and they think that you are trying to teach them their job. -These days doctors don't pay much attention to patients as they mind their own business and less time for patients. - It is up to the Ministry of Health and experts to decide what should be done regarding malaria treatment.	- Find preventive measures against malaria and more research on SP is needed. - More efforts should be done to inform and educate the people about the drug. - There is a need to improve laboratory facilities so that people should be checked for malaria parasites before they are given or prescribed SP. - There is a need to have SP injection formulation. - SP dosage should be reduced especially for under-five children as the drug is too strong though it is a good one. - Radio and newspapers should help in educating people. -SP should be removed from use and chloroquine be brought back -Doctors should be educated on measuring weight of patients before prescribing SP and should check who to give SP.	- All married men take responsibilities as children are our only investment. - Most decisions involving finances are done by men and if the father is not around the wife takes the task in the absence of the husband. - Many women prefer to start with traditional healers for a seriously sick child. -Even if the husband is a drinker, when one gets home we normally ask if things are in order. As the father is a bread earner in most households. - The wife takes care of the children and the father looks for money to feed the family. - Sometimes we men take the responsibility of helping our wives to take a sick child to hospital or go together if we around home. - Most father's can not leave his/her sick child dying without helping each other at home. -We father's participate especially if the sick child is above 5 years and mother's deal with sick under-fives.	- These news people are making business sometime by writing misleading information about SP. - So people need to be careful when reading newspapers or watching television or listening to the radio. - These media do not give or talk about the positive aspects of the drug instead they just talk of the negative aspects of SP. - You will find that most of the side effects of SP we have heard or seen them shown in newspapers, radios and televisions. -Even pictures of affected individuals by SP are shown on televisions and newspapers. - In our village or community we have not yet experienced any person so far getting problems with SP but have only read in newspapers.

In all the FGDs, participants mentioned they had heard about the new drug (SP) on the radio, some few in news papers and the majority were those who had visited health facilities previously and were either informed by the health care workers or they read about it on posters posted on the walls at the health care facilities.

### Experience with "SP"

In the FGDs, most participants expressed a lot of fear and worries about using SP due to reported adverse reactions. Few had actual experience of such reactions, but everyone had heard about people who died after using SP. In one of the FGDs with men, one participant stated that "*hii dawa ni nzuri kwani haina madhara mengi kama watu wanavyosema au kufikiria kwani mimi nimeitumia sikupata madhara yeyote*" [english translation] side effects due to SP use are rare in occurrence and he had used the drug and found it was a good one and never got any side effects (Tables 1, 2, 3).

**Table 3:** Summaries of statements made by different focus groups discussions (4 FGDs) with health workers about SP

**About SP**	**Why switch to SP?**	**How can the situation be improved?**	**What's wrong with the media?**
- Is a drug containing sulfadoxine and pyrimethamine. - We know that some symptoms are caused by SP others not. - Some mothers throw away SP tablets when we give them to administer for their sick children at home. - So we decided to administer SP for all sick children at our facilities under supervision of a health worker. - The drug delays relief of fever symptoms and people interpret it as side effects. - We are not certain whether all these side effects being spoken about are really caused by SP or by other things? These rumours have created a lot of fear. - It is true that SP causes skin rashes, itching, dizziness, headache and some body weakness. However, some people who are allergic to SP may react badly even die if no immediate help is given. - The number of people using SP at our facilities is decreasing daily and as a result we are left with a lot of SP drugs every month. - People claim to get side effects and even leading to sores, loss of appetite, skin colour changes to black as a charcoal in some individuals. - Those who are given SP and they throw away they usually come back the next day or after two days complaining of malaria and that SP did not work. - We try to educate and convince them about the drug, but some clients are difficult to understand	-People think that we are forcing them to use SP. - People believe that as health workers we know everything of what is going on about the drug whether it is being on trial or not. - When patients come to the facility, they choose the doctor they want if we happen to be two or more because they would like to be attended by some one they know they can convenience not to be prescribed SP. - Some of them lie that I am allergic to SP so that they can be prescribed an alternative drug. - Some Members of Parliament have also contributed to people's fear about SP because they have been questioning the Minister for Health about this drug during the parliament session. -Some patients do not seem to trust the water we give them to swallow with the drug for their under-five children.	- Member of Parliament and other ordinary people should be educated about this drug. - Some patients we see suggest to us that the sulphur content should be reduced from SP as they think this is the one which is causing all the side effects and making also the drug strong - Others suggest that chloroquine should be brought back in use. - The Ministry of Health should do more research to find out why the drug is too strong or what makes it be strong and other side effects – The drug needs to be taken together with Paracetamol which is an antipyretic to help reduce the fever. - Educate the news writers or reporters about the drug so that they write proper messages. - Families need counseling especially those who experience or had experienced SP side effects. - Patients who get skin problems as a result of SP we give them hydrocortisone. - We repeat the dose if the patient returns to us complaining of no improvement for the first time since we last gave SP. - The sulphur content should be investigated more by experts. - SP should remain in use but be improved. - Patients should eat and drink a lot of water when taking or after have taken the drug.	- Less educated news writers magnify stories with SP side effects. - These SP stories through media have caused a lot of fear and negative perception about the drug in the general population. - Radio report that SP kills and some newspaper displays pictures of affected individuals and hence causing more fear to people when they see. - Radio and newspaper are most of the time broadcasting problems related to SP with is not based on truth and no positive images about the drug.

Others commented that "SP *ni dawa mbaya sana kwa sababu inaua, inaunguza ngozi mwilini au inababua au kuchuna kabisa ngozi na haishushi homa haraka badala yake inapandisha homa na kwa ujumla ni dawa ambayo ina nguvu sana hasa ukiitumia kwa mtoto au watoto*" [english translation] it is a bad drug as it kills, burns the skin, it does not reduce the fever or temperature and it is too strong especially when used for children.

Furthermore, during the FGDs, some participants with mothers and men were very angry and thought they were part of an experiment staged by the Tanzanian government to see how the drug kills or how many people would be killed by using SP [Swahili translation] "*serikali yetu imeamua kufanyia majaribio hii dawa ya SP kwetu ili waone kama watu wangapi watakufa*" (Tables 1 &2).

### The mass media

Participants in all FGDs, had heard about severe adverse reactions through mass media, most commonly newspapers and radio. Several participants suggested that "*waandishi wa habari lazima waelimishwe namna ya kuandika habari zinazohusu madawa vizuri hasa dawa hii ya SP kwani wanavyoandika kwenye magazeti na watu wakasoma na kuona picha ya mtu aliyeunguzwa na dawa hii kila mtu anaogopa na kusema hataitumia hii dawa*" [english translation] the news media or writers should be educated about SP drug and how to write proper medical news or information in the correct way which will not scare the audience when they read. They went further to say information is very important as it may build or destroy the image of the drug. Once people have read or heard about it, it is difficult to change their mind back (Tables 1, 2, 3).

### Healthcare workers observations and experience

Because of the fear and negative perception about SP, all FGDs with health workers explained "*kwa sababu ya kuona watu wanaiogopa hii dawa na akina mama wengi wakipewa wakampatie moto SP nyumbani wengine wanaitupa njiani hivyo tumeamua watoto wote wenye homa tunawapatia SP wameze hapa hapa hospitalini na tunamwangalia moto kwa muda wa nusu saa, kama hajatapika ndipo tunamruhusu mama aende na moto nyumbani na akitapika tunarudia tena kumpatia dawa*" [english translation] they had to give the drug under observation in the health facility to avoid mothers throwing away the drug (SP tablets) on their way home instead of giving them to their sick children (Table 3). To combat this practice, the child was made to swallow the drug in the presence of a healthcare worker and was observed for 30 min before being allowed to go home in order to make sure the child did not vomit the drug. This practice of the healthcare workers was confirmed by FGDs with mothers. The DOT for SP in under-five children was applied in these facilities and if the child vomited within this period another dose was administered by the health worker (Table 1).

## Discussion

Sulfadoxine-Primethamine (SP) was previously reported to be too strong for children in the same district [10] and that the drug had characteristic properties of rapidly clearing parasites but slow fever clearance [15]. From the FGDs it was revealed that people knew that SP was available and was used as a first line drug for treatment of malaria. However, other FGDs reported that the drug (SP) increases fever and makes the general body weak after taking the drug.

In spite of the described negative perceptions about SP, the drug was used in these communities. However, experience from African countries like Malawi and Kenya which introduced SP as first line treatment shows that people continue to use chloroquine after the introduction of the new drug due to scepticism about the efficacy of the new drug or fear that "a potent" drug could be dangerous [16]. With such a change in anti-malarial drug policy, mothers/guardians had no option except to seek care at the public health care facilities. However, this health seeking behaviour by mothers /guardians was accompanied with other consequences related to personal, social and economic problems. For example walking distances and long waiting time must be considered as factors which may affect treatment. These delays prevent children from getting early diagnosis and prompt treatment with first line anti-malarials as advocated by the Roll Back Malaria (RBM), the Integrated Management of Childhood Illnesses (IMCI) and Home Based Management of Fever (HBMF) [17].

In general our results from the FGDs with mothers/guardians and men revealed some good awareness about SP despite the fact that some of them were hesitant in accepting the drug. All these fears, negative perception about the drug are due to several factors such as; its slow pharmacological effects or actions in providing quick relief of fever symptoms, instead it takes up to three days to resolve the fever. Secondly, because of the reported rare but serious side effects which occur to some individuals being reported through mass media. This rare side effect is medically known as Steven's Johnson's Syndrome.

Futhermore, FGDs with mothers and men gave strong statements about the drug such as "our government has decided to use this drug (SP) for experimental or research purposes so that they can see how it kills or how many people will be killed after using this drug. They went on further to say "SP is a bad drug as it kills, produces adverse skin reactions, does not reduce the fever, and instead raises, and was too strong for children". These are strong allegations to the government (Ministry of Health) which are not true per se because no government would subject her people to a harmful drug. These comments show how some groups of participants were illiterate or not well informed about the drug (SP) properties or actions.

A concern regarding SP raising fever for children was discussed on several occasions in the FGD groups. The other negative effects about the drug included fear of side effects, burning the skin or producing blisters. Thus, the drug was negatively perceived in that way. Some FGDs with mothers/guardians clearly reported that they were forced to use SP in public health care facilities. Nevertheless, they used the drug because chloroquine was no longer available. In their opinion, they would have liked to continue using CQ as they believed the drug was still effective in treating malaria.

FGDs with healthcare workers reported using a direct observation therapy (DOT) when administering SP to the sick under-five children in health care facilities. This DOT approach was stated also by mothers FGDs. This DOT approach is important because mothers who fear and have negative impressions of the drug can not administer the given drug at home. Instead they may give an inefficient substitute or not give anything to the sick child. This is a dangerous practice as it allows the disease to progress from mild to severe forms. The severe form of the disease for a sick under-five child in most cases is accompanied with fatal consequences or outcomes such as cerebral malaria, anaemia or death. Sadly, you may find these mothers coming back to the facilities on the following day or after one to two days complaining that the drug did not work while in fact they never administered it to the sick child.

Previous studies reported lack of anti-malarials at public health care facilities especially during the chloroquine era [18,19]. However, SP was available and free of charge for all sick under-five children in Tanzania. The possible reason why SP was available as reported by FGDs with healthcare workers was because they received the same number of tins (quantities of SP) as during the chloroquine era in public facilities. SP is given as a single dose as compared to chloroquine which was administered spread over 3 days. The anti-malarial fixed combination therapy (Coartem) which Tanzania will be changing to early next year (2006), has the dosage schedule spread over 3 days like chloroquine which may compromise compliance. Perhaps it is important to consider and plan to order more tins of the new drug as it will be given on a 3 day regimen. Otherwise, we could repeat the problem previously experienced with chloroquine. That is the new drug (Coartem) being not readily accessible and available all the time in most primary health care facilities in the country.

However, SP is expensive as it costs 5–11 times the price of CQ [10]. If this drug was not given free at the public facilities, the poor Tanzanians, especially in rural communities, would not have been able to purchase the drug and this would result in poor treatment seeking behaviour for malaria [20-22]. Due to fast development of resistance to SP, Tanzania will soon be moving from SP (monotherapy) to an expensive fixed combination anti-malarial therapy that is ten-times more expensive than SP. A full course or dose of artemether-lumefantrine (Coartem) will cost 6,000–10,000 Tanzania shillings (equivalent to 6–10$). Initially the drug will be given free of charge for a period of about 3 years or more through the Global Fund (GF) support. Thus, this calls for concerted efforts to be put in place by the National Malaria Control Program within the Ministry of Health to properly educate the people on rational practices of using this drug once it becomes officially available. Rational use of this fixed combination drug will help to prolong the life span of this expensive medication.

SP was reported to be in use through FGDs in spite of the reported fear and wide spread negative perceptions about the drugs side effects. Thus, any future anti-malarial drug treatment change in the country, should consider the practical aspects of community involvement and acceptance of any new anti-malarial drug to be introduced. The implementation phase should focus more on community sensitisation of the people using various communication approaches or strategies such as use of mass media, news, radio and television. These communication strategies should bear correct, relevant, and short clear messages. Sensitizing and educating the people to raise their knowledge and awareness is of paramount importance for the success of the program.

However, the commonest reported media were newspapers and radio and on rare occasion television was mentioned. Nevertheless, some participants commented that there was a need for educating news writers about the drug (s), in order for them to write proper messages about the drug. Wrong messages are likely to divert attention of the people in the wrong direction and hence create a lot of fear with an increased negative perception about the drug in use or to be used. Thus, there is a need for both intervening and doing more research in this area of assessing the quality of messages sent out to consumers, the way messages diffuse out in the rural communities of Tanzania and the way they are perceived if we are aiming to improve future communication strategies for any new anti-malarial drug policy change to follow early next year in Tanzania.
